# A Volume Measurement Method for Lunar Soil Collection Based on a Single Monitoring Camera

**DOI:** 10.3390/s18103394

**Published:** 2018-10-10

**Authors:** Shaowen Ding, Xiaohu Zhang, Qifeng Yu, Lichun Li, Jie Wang

**Affiliations:** 1 College of Aerospace Science and Engineering, National University of Defense Technology, Changsha 410072, China; dswen0611@126.com (S.D.); ufg@vip.sina.com (Q.Y.); lichunmail@163.com (L.L.); wangjie93nn@sina.com (J.W.); 2 School of Aeronautics and Astronautics, Sun Yat-Sen University, Guangzhou 510000, China; 3 Beijing Aerospace Control Center, Beijing 100000, China; 4 Beijing Institute of Space Mechanics & Electricity, Beijing 10000, China

**Keywords:** volume measurement, terrain reconstruction, camera orientation, point cloud registration, change detection, point cloud comparison

## Abstract

In the task of lunar soil collection, estimating the volume of the collected soil is an important part of the sampling control of the lander. Due to the design constraints of the lander, there is no additional installation position for volume measurement equipment. To fully use the sensors already installed, a collected soil volume measurement method is designed in this paper based only on a single monitoring camera. This method uses a sequence of images of the collection area captured by the camera mounted on the acquisition arm to accurately reconstruct the terrain of the collection area surface before and after soil acquisition. Additionally, bi-temporal dense point clouds are reconstructed. Based on the area of change associated with soil collection, the constructed dense point clouds are compared according to the topographic characteristics of the area to estimate the volume of soil collected. Experiments show that the method is stable and reliable and can meet the requirements of actual measurement tasks.

## 1. Introduction

Soil collection is an important task for lunar landers working on the moon. Researchers often want to estimate the collected soil volume during sampling tasks, and weighing is the most direct way of doing so. However, due to the constraints of the lander design, quality restrictions, etc., quality measuring equipment is usually not specifically installed for these types of measurements [1]. With the continuous development of 3D reconstruction technology, it has become feasible to indirectly calculate the collection amount by analyzing the variation in the three-dimensional shape of the soil collection area. The sensors currently available for reconstructing small-scale 3D terrain include structured light cameras, time of flight (TOF) cameras, and visible light cameras. However, installing a structured light camera or TOF camera on a detector can also increase the design difficulty of the lander. Additionally, the accuracy of a TOF camera can only reach the order of cm [2], which often fails to meet the measurement requirements. Because a large number of visible light cameras are distributed on the lander, if they can be effectively used, it is possible to reconstruct the collection area before and after soil acquisition using 3D reconstruction technology. Then, the soil area can be analyzed by comparing the results of the reconstruction to estimate the amount of collected soil.

To achieve the above objectives, the correct image acquisition device must be chosen. Most lunar landers are fixed with navigation cameras, panoramic cameras, and obstacle avoidance cameras [3]. Navigation cameras and panoramic cameras are not suitable for terrain reconstruction due to the associated installation angle and functional design constraints. The obstacle avoidance cameras are binocular cameras fixed under the lander that can be used to reconstruct three-dimensional terrain in front of the lander based on binocular stereomatching technology. This technology can detect the lunar surface topography and assist in obstacle avoidance. However, analyses have indicated that the reconstruction result is severely restricted by the field of view of the camera and that the reconstruction range is limited to the overlapping field of view of the binocular cameras. Moreover, the reconstruction results will have gaps because of occlusion problems caused by topographic relief [4]. Therefore, it is not appropriate to measure the collected soil amount using a fixed binocular camera.

In addition to the cameras mentioned above, a monitoring camera is usually installed on the soil acquisition arm, and it can provide images of the collection area. It has the advantage of flexible visual angle selection and is not limited by field constraints. A sequence of images captured by the monitoring camera comprises a group of overlapping images taken from different angles in the soil collection area, and the images span the entire area to be measured. Theoretically, the surface morphology of the collection area can be completely reconstructed, and the collected soil volume can be accurately measured by analyzing the reconstruction results. Subject to the constraints of the relevant tasks, very little practical work has been conducted in this context. Therefore, to investigate the above concepts and ensure that the design scheme is feasible, it is necessary to analyze the various aspects of collection area reconstruction and compare the reconstruction results.

This paper uses the sequential images of the collection area captured by the monitoring camera and designs and implements a method for measuring the amount of soil collected by the lunar detector. There are two key problems addressed with this method: one involves completely and accurately reconstructing the collection area, and the other includes comparing the reconstruction results before and after soil acquisition to determine the volume of the collected soil. A method is proposed and refined based on these two problems.

## 2. Multiview Reconstruction for the Soil Collection Area

### 2.1. Camera Motion Trajectory Analysis and Reconstruction Process Design

**Figure 1:**
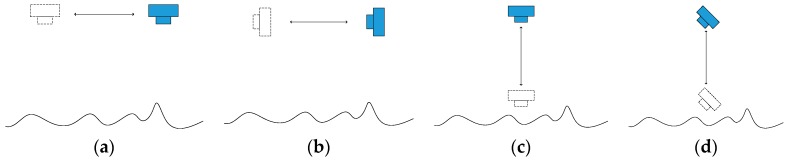
Image acquisition trajectories of monitoring cameras. (**a**) Translational motion, camera downward viewing; (**b**) Translation motion, camera tilt; (**c**) Vertical motion, camera downward viewing; (**d**) Vertical motion, camera tilt.

As discussed above, the collected soil volume can be obtained by comparing topographic reconstruction results, and one of the key processes is completely and precisely reconstructing the collection area. The camera position and attitude considerably influence the results of multiview reconstruction. Unlike in a ground test, a camera fixed on the mechanical arm of a lander cannot be controlled to obtain the best position and angle according to the research needs, and these factors are limited by the inherent trajectory of the acquisition task. In a soil collection task, there are four common types of camera trajectories, as shown in Figure 1.

Due to the influence of terrain undulation factors, the images taken in situations (b) and (d) may contain many occlusions that can create gaps in the reconstruction results. Therefore, these trajectories are not suitable for the subsequent point cloud comparison and volume calculation. In the case of type (c), as well as type (d), the intersection angle of the area directly below the camera is too small, which will result in low reconstruction accuracy. Obviously, type (a) is the most suitable for collection area reconstruction. In the actual image acquisition process, the camera cannot be controlled to fully move based on the ideal trajectory. Therefore, this factor should be considered in the subsequent method design, and inappropriate images should be removed from the sequence of images.

**Figure 2:**
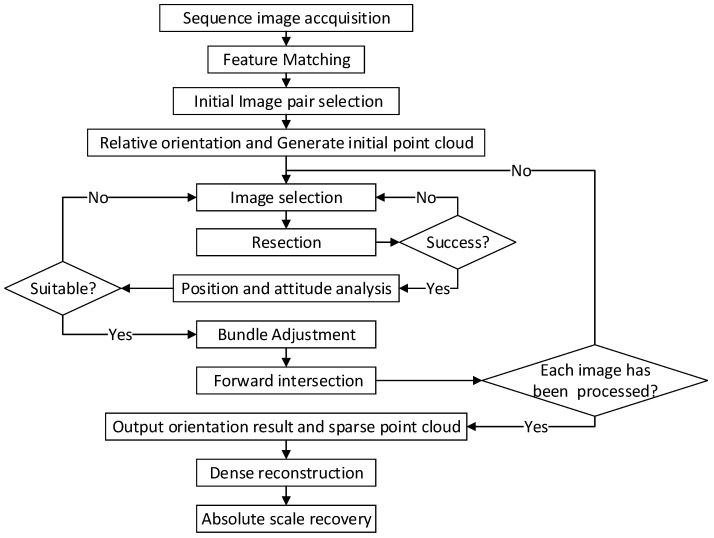
Multiview reconstruction process of the soil collection area.

The basic 3D reconstruction process of the soil collection area is designed as shown in Figure 2. Because there are mature algorithms currently available for feature matching and dense reconstruction, they are not the emphasis of this paper. The high-precision orientation of the camera at each moment is one of the most important aspects of multiview reconstruction, and it directly determines the accuracy of the final reconstruction result. Therefore, this section focuses on the high-precision orientation of the camera for reconstructing the collection area, and all images used for reconstruction should meet the relevant trajectory requirements. An incremental method has been designed according to the characteristics of the acquired sequence of images analyzed above to subsequently calculate the associated collection amount.

The internal parameters of the fixed-focus camera are calibrated in advance. A sequence of images of the area is obtained from multiple angles, and these partially overlapped images are then utilized. Because many images are obtained, it is necessary to design a reasonable process based on image registration to ensure the reliability and efficiency of the algorithm [5]. To perform registration between images, features must be extracted from each image; specifically, the features of two images must be matched, and RANSAC (Random Sample Consensus) [6] is used to eliminate mismatches. Considering the specific trajectory of the camera, there may be a scaling relationship between adjacent images in the overlapping area. Therefore, SIFT (Scale-invariant Feature Transform) [7] is selected as the feature detector because it has favorable invariance for image scaling. By analyzing the matching relationships between feature points in images, the relevant matching links can be obtained. Each matching link consists of feature points that correspond to the same point in space. With these matching links, the relations among all feature points can be described, and the relative orientation information associated with the cameras at all moments can be obtained according to the following steps.

### 2.2. Initial Image Pair Selection and Initial Point Cloud Acquisition

First, two initial images are selected for relative orientation assessment and forward intersection to obtain the initial point cloud of the feature points. The initial point cloud is used for resection and other images determine the camera position and orientation at each moment. The newly oriented camera information is included in the forward intersection calculation to expand the point cloud. By iterating the above process, the camera position and orientation can be obtained for the entire sequence of images, and the positions of all feature points in the initial point cloud coordinate system can be determined. The initial image pair is used to determine the initial point cloud position, and the initial point cloud is the basis for subsequent camera orientation calculations. Therefore, in the process of camera orientation for the surface reconstruction of the collection area, the selection of the initial image pair is very important.

**Figure 3:**
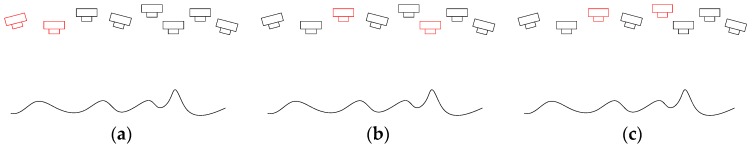
Choosing cameras corresponding to the initial image pair based on different methods. (**a**) The initial image pair is not closely related to the positions of other moments’ cameras; (**b**) The position between the cameras of the two moments corresponding to the initial image pair is too far; (**c**) Appropriate choice.

The positions of the cameras at each moment in the lunar soil collection task are almost distributed in a straight line. In this case, the selection of the initial image pair will greatly affect the orientation accuracy of all cameras. The original method involves directly selecting the two images that have the most matching pairs. However, if the positions of the two moments’ cameras which corresponding to the initial image pair is not closely related to the positions of other moments’ cameras, as shown in Figure 3a, cumulative errors will be introduced in the process of sequentially orienting the cameras. This error can notably reduce the accuracy of all related calculations.

Most of the methods used to solve the above problems introduce the concept of matching link’s length [8]. If K represents a matching link of the sequence images, then K’s length is the number of all the feature points that correspond to the same spatial point p_k_, and this value is recorded as *L_k_*. If *L_k_* is large, it means the spatial point is associated with many images. In the previous literature [8], each pair of images were chosen to be analyzed, and if the number of matching pairs in the two images is larger than the specified threshold, the sum of the matching link’s lengths in the two images will be calculated. All the results are then sorted, and the two images with the largest results are the initial image pairs. This method can achieve satisfactory results; however, subsequent studies have found that the above results may not be optimal image pairs in some cases, as shown in Figure 3b, in which the overlapping area of the two images is small and the number of points in the initial point cloud obtained by the intersection is not sufficiently large. Therefore, this approach is not conducive to improving the orientation precision of other cameras.

Based on this deficiency, this paper improves the initial image pair selection method. In this case, U_*i*_ represents the average matching link length for all the feature points in image *i* and can be expressed as follows:

(1) $$U_{i}=\frac{1}{M_{i}}\sum_{k=1}^{M_{i}}L_{k,i}$$

where M*_i_* represents the number of feature points in image *i*. Taking two images p and q, the feature point pair relationships are used to determine the relative orientation. Then, the intersection angle of each matching pair is calculated, excluding the matching pairs with resulting values that are less than the intersection threshold (generally set to 2°). Next, record the number of remaining matching pairs as F*_p,q_*. The following formula can be used to determine the initial image pair.

(2) $$J_{p,q}=\alpha \cdot \frac{M_{p}U_{p}+M_{q}U_{q}}{U_{\max}\cdot (M_{p}+M_{q})}+\beta \cdot \frac{F_{p,q}}{F_{\max}}$$

The first term on the right side of the above equation describes the degree of association between the image pair (*p,q*) and other images in the sequence, and the second term describes the degree of association between images *p* and *q*. J*_p,q_* is the weighted fusion result of the two terms. J is calculated for each pair of images, and the results are sorted. The two images with the maximum values are selected as the initial point pair. This method not only ensures that the image pairs are closely related to other images, but also ensures that there are many common feature points between the two images, as shown in Figure 3c.

After determining the initial image pair, the relative position and orientation information must be obtained. This information includes the rotation matrix ***R*** and translation vector ***T*** between the two cameras. Many relative orientation methods [9,10,11] can be used in this step. For the convenience of description, hereafter in this text the two images and the related cameras are called left and right image, left and right camera. The specific methods for determining the relative position and posture of the left and right cameras are as follows [12]:

(3) $$\left|\begin{array}{ccc} t_{x} & u & r_{1}^{T}u^{\prime }\\ t_{y} & v & r_{2}^{T}u^{\prime }\\ t_{z} & 1 & r_{3}^{T}u^{\prime } \end{array}\right|=0$$

In the formula, $T={\left[t_{x},t_{y},t_{z}\right]}^{T}$ is the direction vector from right camera’s optical center to the left camera’s, $u={\left[u,v,1\right]}^{T}$, $u^{\prime }$ are the normalized coordinates of the feature points in the left and right images, $\left\{r_{i}\right\}$ is the ***i*** column vector of the rotation matrix ***R*** between the left and right cameras. Each feature matching pair can provide a constraint equation ***fi***, simultaneous equations can be obtained from multiple matching pairs, rotation matrix ***R***’s three angles and vector b are parameters to be estimated. Since b is a directional vector, actually it contains only two unknown parameters. So there are a total of five unknown parameters to be calculated, and five feature matching pairs are enough for getting an analytic solution of the simultaneous equations above theoretically [12]. In fact, the feature matching pairs are far more than five groups. In order to obtain accurate relative orientation information, linearize the constraint equations ***fi*** by use of first-order Taylor expansion, then achieve the optimal solution by using Gauss–Newton or Levenberg–Marquardt iterative optimization method to minimize the objective function $F=\sum_{i}f_{i}^{2}$. The coordinate system of the left camera is set as the reference system, and the position and orientation of the right camera are determined based on the reference system. This step yields the relative orientation of the initial image pair.

Because the internal and external parameters of the first two cameras are known based on the above steps, the initial point cloud can be constructed using the forward intersection method to determine the spatial coordinates of the points corresponding to the feature matching pairs. The precision of the initial point cloud influences whether the subsequent algorithm can be effectively executed. Inaccurate calculations may cause failure during the resection step. Therefore, it is necessary to remove the points with large error from the point cloud. In the process of binocular intersection, an inappropriate intersection angle (too large or too small) will greatly reduce the precision of calculating the spatial positions of intersection points. The solution involves a screening procedure in which the intersection angle of each point is calculated, and if the angle is less than the threshold *θ*° or larger than (180 − *θ*)°, the point is removed. Experiments have shown that setting *θ* to 2 can meet the accuracy requirements of most situations. If the number of feature points is sufficiently large, the threshold value can be increased to obtain higher precision.

### 2.3. Camera Orientation at Each Moment and Dense Point Cloud Computation for the Collection Area

After the calculation of the initial point cloud, the positions and orientations of other cameras can be determined by the resection method. The other images are ranked based on the number of corresponding feature points related to the initial point cloud, and the image with the most points is selected for resectioning. This is only the initial value calculation, and the position and orientation are eventually optimized. Therefore, the PNP monocular estimation algorithm can meet the accuracy requirements of this approach [13,14]. The algorithm requires at least 6 points [15], and the number of feature points is far greater than that in most cases. As long as there are certain overlapping regions in adjacent images from the sequence, the position and orientation estimation conditions can be satisfied. After determining the relative position and attitude relationship between the camera at the current moment and those of all other oriented cameras, it is necessary to assess whether the image corresponding to the camera at the current moment meets the reconstruction requirements discussed in the conclusion of Section 2.1. If the position and orientation conditions of a camera are not satisfied, the corresponding image of the moment must be deleted from the sequence.

After completing the resection tasks, for the camera corresponding to the image, forward intersection with other oriented cameras is used to expand the point cloud. To control the cumulative error caused by the backward transmission of computational error, the position and orientation parameters of each camera at each moment in the point cloud should be optimized. Here, bundle adjustment is used to perform the optimization. The sum of the reprojection error for each point in each image is taken as the objective function, and the position of each point in the point cloud and the camera orientation at each moment are the parameters to be optimized. At this time, the orientation of the third image is determined, and the sparse point cloud is expanded. Next, the subsequent image is selected and oriented, and the above steps are repeated until the camera has been oriented at every moment based on the reference system.

**Figure 4:**
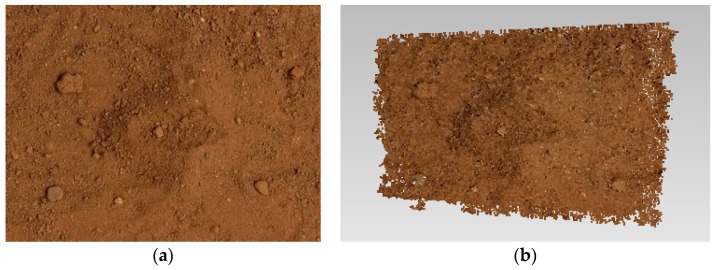
The reconstructed dense point cloud of the collection area. (**a**) Collection area image; (**b**) Reconstruction effect of the corresponding area.

At this time, the camera at each moment has been accurately oriented based on the steps above. Based on the orientation calculation results, the dense point cloud of the collection area, which includes the 3D information from the surface, can be obtained using a stereovision method [16]. The basic concept is that for any pixel in any image, the corresponding position in adjacent images can be located by the patch matching method. Then, the spatial position of the pixel can be obtained by intersection. Processing all the pixels in a sequence of images yields the positional fusion of all corresponding spatial points to obtain the final dense point cloud. Many mature methods have been designed based on this concept, and there are many slight differences in the proven application and actual effect of various methods. Here, Zhu’s robust high-precision and occlusion multiview stereovision method [17] is selected to rebuild the dense point cloud of the soil collection area because of its high performance and ability to overcome occlusion effects. It can be seen from Figure 4a that there are slight occlusions at the bottom of some small stones at some angles, but this is unavoidable. Many multi-view dense reconstruction algorithms can’t reconstruct this part completely, and Zhu’s method can get better results. Based on the accurate camera orientation data obtained above, the desired dense point cloud can be effectively reconstructed. A sample of the results is shown in Figure 4b.

In the process of camera orientation determination, the scale factor is not considered, so there is a scale factor difference between the reconstructed result and the actual area. To obtain the reconstruction result at the actual scale, the available scale datum should be established in the collection area, and the scale factor can be calculated from the ratio of the reconstruction distance to the actual distance between reference points.

In soil collection tasks, it is unnecessary to place a real scale datum at the collection area. In fact, the motion information for the acquisition arm can be provided by motion sensors installed on the arm, and the position of the camera’s optical center relative to the collection arm is always the same. As long as the relative positional relationship between the camera’s optical center and the acquisition arm has already been calculated on earth by hand-eye calibration method [18], the actual movement distance of the camera’s optical center between any two image acquisition moments can be obtained. Because the position of the optical center in the reference system has already been accurately determined during orientation, the movement distance of the optical center between two moments can be regarded as the virtual scale datum and used to calculate the scale factor. Based on the calculated scale factor, the reconstructed dense point cloud of the collection area can be scaled to the actual size.

## 3. Soil Collection Estimation by Point Cloud Registration and Comparison

The local collection area changes after soil collection. Therefore, to measure the volume of the collected soil, it is necessary to evaluate the change in the morphology of the collection area. According to the steps presented in the previous section, the dense point cloud of the surface of the collection area can be reconstructed. The dense point cloud is reconstructed before and after soil collection as *C*_0_ and *C*_1_, respectively. Then, the volume calculation problem is converted to a point cloud registration and comparison process involving the bi-temporal dense point clouds [19].

### 3.1. Registration of the Reconstructed Point Cloud

**Figure 5:**
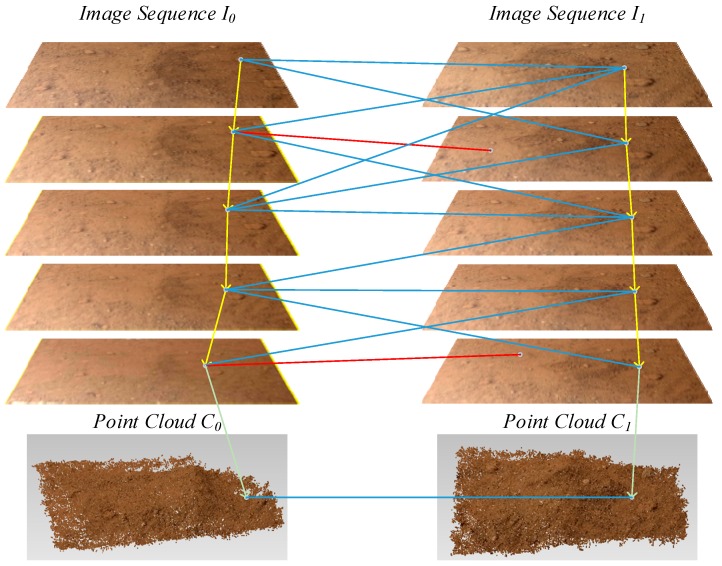
Registration of reconstructed point clouds in the collection area.

The registration of reconstructed point clouds before and after soil collection is one of the core steps in the soil measurement process through point cloud comparison and is the basis for subsequent calculations. The main objectives of point cloud registration are to calculate the projective transformation matrix using point correspondence and to unify *C*_0_ and *C*_1_ in the same coordinate system. Using 3D local feature descriptor to detect and match the 3D feature points is the common method to obtain the point correspondence between the two point clouds. However, since the soil collection will bring changes to the collection area, and the two sets of sequence images (noted as *I*_0_ and *I*_1_) used to reconstruct *C*_0_ and *C*_1_ have different image acquisition paths, there must be many differences between the two reconstructed point clouds. Moreover, because there are errors in the reconstruction process, the two point clouds’ common areas are not exactly the same at the detailed local scale. This adds difficulties to the registration based on 3D feature points. To overcome this issue, another method is designed for point cloud registration. During the process of camera orientation, a sparse point cloud composed of feature points is simultaneously obtained. The dense point cloud and sparse point cloud are rebuilt in the same coordinate system. If the feature points in the unchanged area match in images *I*_0_ and *I*_1_, then the point correspondence between the sparse point clouds before and after soil collection can be obtained, and *C*_0_ and *C*_1_ can be registered. The specific steps in this process are shown in Figure 5.

**Figure 6:**
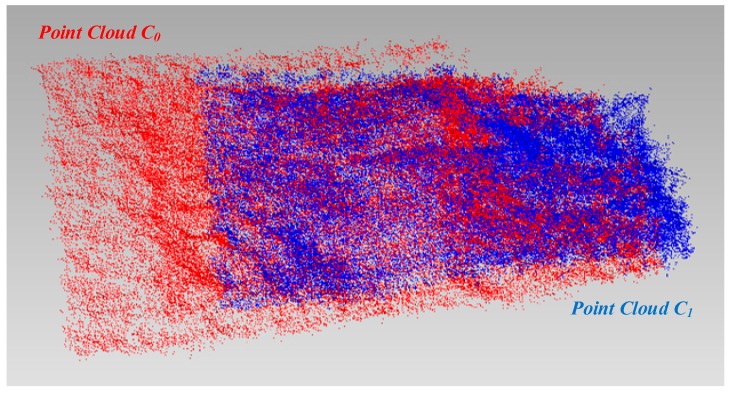
Registration result for the bi-temporal dense point clouds of the soil collection area.

First, each image in the image sequence *I*_0_ is matched with all the images in sequence *I*_1_. Because the image acquisition areas of the two sequences are identical, the matching results contain a large number of correct matching pairs, as well as false matches. For any matching link *ml*_0_ in *I*_0_, the link corresponds to a spatial point in the sparse point cloud of the collection area before soil collection. The corresponding matching points of *ml*_0_ are analyzed for each node (feature point) in all the *I*_1_ images to determine whether there is a matching link *ml*_1_ corresponding to *ml*_0_ in *I*_1_ that satisfies the condition that the majority of the matching points among the nodes of *ml*_0_ in sequence *I*_1_ are nodes of *ml*_1_ (the proportion is higher than the specified threshold). If this condition is true, then take all the nodes of *ml*_1_ analyzed, and a reverse verification method is used to determine whether *ml*_0_ is the corresponding matching link for *ml*_1_ in image sequence *I*_0_. If the result is the same, the node points of *ml*_0_ in *I*_0_ and the node points of *ml*_1_ in *I*_1_ correspond to the same points in space. Through these steps, a matching pair is identified between the *C*_0_ and *C*_1_’s coordinate systems. Next, all the matching links of *I*_0_ are investigated to obtain more pairs of spatial matching points. RANSAC is used here to estimate the rotation matrix and translation vector between the two point clouds with these matching pairs, and the exact solution can be obtained by using the iterative closest point algorithm (ICP) [20]. The final rotation matrix and translation vector can be used to combine *C*_0_ and *C*_1_ into one coordinate system. Figure 6 shows the registration result for the reconstructed point clouds of the collection area before and after soil collection.

### 3.2. Volume Calculation by Comparing Point Clouds

#### 3.2.1. Confirm the Change in Area Caused by Soil Collection in the Point Cloud

**Figure 7:**
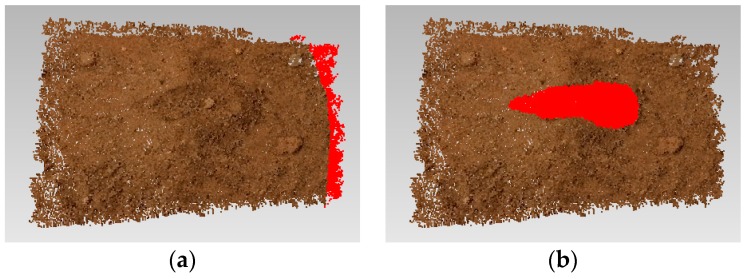
Differential region in *C*_1_ compared with *C*_0_, (**a**) Caused by a trajectory difference; (**b**) Caused by soil collection.

The soil collection information is included in the difference between the two clouds *C*_0_ and *C*_1_. First, the two clouds must be compared to find the difference caused by soil collection. A method of determining the differential region is proposed here. First, a sensitivity indicator *Q* is defined for the differential region:

(4) $$Q=\sum_{\Omega }{\left\|p_{0}^{i}-p_{1}^{i}\right\|}_{2}$$

where *p*_0_ is a point in *C*_0_ and *p*_1_ is the closest point to *p*_0_ in *C*_1_. In the *p*_0_-centric small region Ω, the sum of the distance between each point and the nearest point in *C*_1_ is calculated. If the calculated value is larger than the specified threshold, point *p*_0_ has an obvious change in position before and after collection. Each point in *C*_0_ is traversed to find all the points with obvious changes, and isolated points are removed. Then, the difference between *C*_0_ and *C*_1_ is determined. The differential region above can be divided into two parts. One part is associated with the different trajectory of the camera, and the other is the morphological change due to soil collection. Because the former is distributed at the edges of the reconstructed area, the two parts are easily distinguished, and the area of change associated with soil collection can be identified, as shown in Figure 7.

#### 3.2.2. Comparison of Dense Point Clouds in the Collection Area

**Figure 8:**
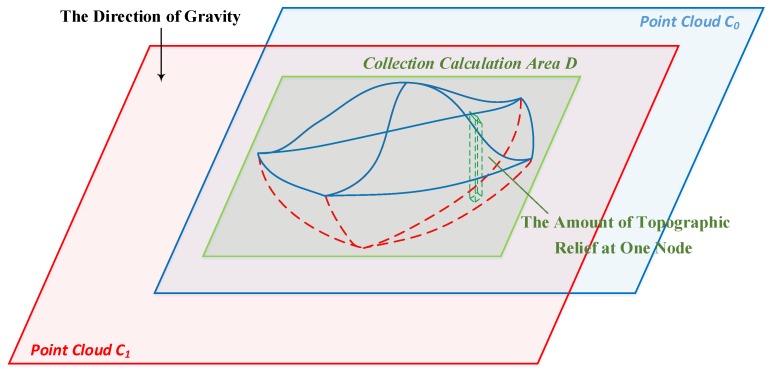
Soil collection calculation based on the cumulative relief.

It is difficult to directly calculate the volume change by comparing the changes in the two point clouds, and no practical method is available for such an analysis. Although a mesh comparison may provide a feasible solution, the result may be greatly influenced by the generation accuracy of the mesh, which can introduce additional errors. Because the collection volume information is included in the change in topographic relief, the required value can be obtained by measuring the relief at each position in the changed region and summing the topographic relief differences over the entire area. Based on the above concepts, a method is designed to calculate the volume of collected soil by comparing point clouds. Because the differential region in the collection area is irregular and the reconstructed point cloud is discontinuous, the method is implemented using resampling and difference accumulation methods to ensure satisfactory accuracy. A schematic diagram of the process is shown in Figure 8.

In this method, the first step involves estimating the direction of gravity for the entire rebuilt terrain based on the reconstructed dense point cloud. Then, a two-dimensional collection area *D* is identified based on the location of the differential region associated with soil collection. The area *D* is discretized to determine the topographic relief value at each node *pij* in *D* by calculating the distance between the resampling points of *C*_0_ and *C*_1_ in the direction of gravity. Then, the topographic relief is calculated for all nodes and summed to obtain the total amount of collected soil.

The reason why the gravity direction is determined first is that the resampling points of the two point clouds *C*_0_ and *C*_1_ in this direction must have a one-to-one correspondence because of the influence of gravity. Considering the flat distribution of the reconstructed terrain point clouds, the gravity direction of the terrain is roughly the same as the direction of the principle axis of inertia of the point clouds. Therefore, we use the principal component analysis-based approach to approximate the gravity direction [21,22]. The direction of the principle axis of inertia is determined by the inertia tensor *I* of the dense point cloud.

**Figure 9:**
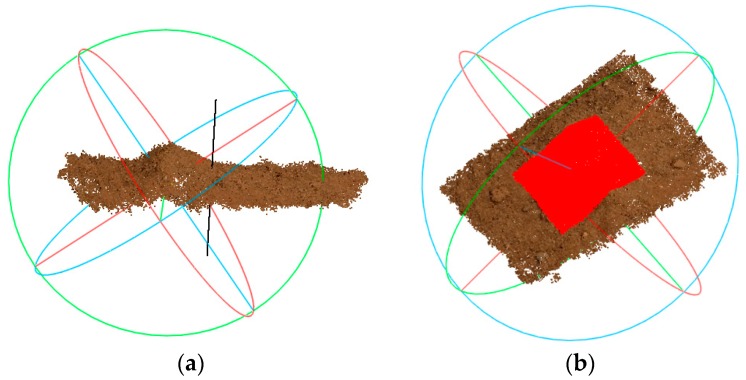
The gravity direction and collection calculation area based on the reconstructed point clouds. (**a**) Estimation of the gravity direction; (**b**) The collection calculation area *D*.

Then, the eigenvectors of *I* are determined, and the gravity direction $\vec{l}$ is the eigenvector corresponding to the minimum eigenvalue, as shown by the white line, and corresponding direction, in Figure 9a.

The change in the area of the point cloud was discussed in Section 3.2.1, and the reconstruction algorithm leads to density differences in the two point clouds at each position. Therefore, it is necessary to determine the collection calculation area *D* for resampling. First, the area is discretized, and resampling is performed at each node. A subsequent comparison of *C*_0_ and *C*_1_ is performed for each sample node of *D*. For the convenience of discretization and to include all nodes in the region, *D* is set to a rectangular area in a spatial plane. The plane is determined by the gravity direction $\vec{l}$ and the gravity center of point cloud *C*_0_. All the points in the collection area of *C*_0_ are projected to the plane, and the four vertices of the rectangular region can be determined by the extremum coordinates of the projected points. In this manner, the point cloud comparison based on area *D* can cover the entire area of change. The length and width of the regional *D* are expressed as *a* and *b*, and the step length of resampling is set to *s*. The number of sampling nodes in area *D* is (*a*·*b*)/(*s*·*s*). *D* is shown as the red area in Figure 9b.

For each resampling node *p_ij_* in area *D*, a line *L_ij_* is identified that includes point *p_ij_* in direction $\vec{l}$. The closest point to *L_ij_* in *C*_0_ is determined, and the point is rotated about *L_ij_* to obtain *m_ij_*, as the sampling point of *C*_0_ associated with the current node. Additionally, the resampling point *n_ij_* of *C*_1_ at node *p_ij_* is obtained.

Next, the distance between *mij* and *n_ij_* is multiplied by the square of the sampling step length *s*, and the result is the variation between *C*_0_ and *C*_1_ at the current node. The volume *M* of collected soil can be converted to the sum of the variation at all discrete nodes in area *D*. The associated formula is as follows.

(5) $$M=\sum_{i=0}^{a/s}\sum_{j=0}^{b/s}\left(s^{2}\cdot {\left\|m_{ij}-n_{ij}\right\|}_{2}\cdot \frac{mz_{ij}-nz_{ij}}{\left|mz_{ij}-nz_{ij}\right|}\right)$$

## 4. Simulation Experiment Involving a Volume Measurement of Lunar Soil Collection

**Figure 10:**
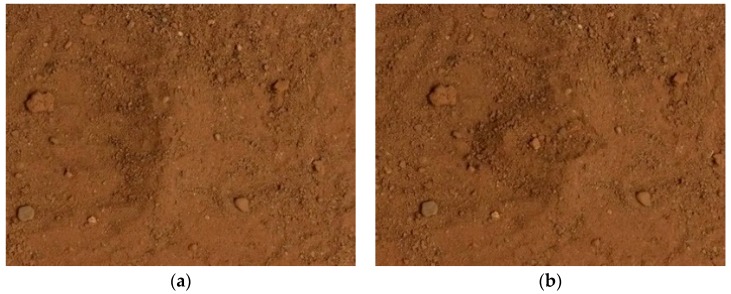
Simulation of lunar soil collection. (**a**) Before collection (vertical view); (**b**) After collection (vertical view).

The following simulation experiment on the ground was conducted to verify the feasibility of the proposed method. The simulated lunar terrain area is approximately 500 mm × 800 mm × 20 mm, and a small collection shovel was used to simulate the collection process. Using an ordinary fixed-focus microcamera to capture images. The overlap ratio is controlled at more than 50%. the image resolution was 640 × 480 pixels, which is consistent with the actual parameter. Example simulation images are shown in Figure 10.

Camera movement was controlled according to a predetermined trajectory, and a sequence of images of the region was obtained from multiple angles.

### 4.1. Comparative Experiments for Camera Orientation and Point Cloud Registration

In order to verify the accuracy of the camera orientation, the re-projection error [23] is calculated after the bundle adjustment. Several methods such as SFM (Structure from Motion) [24], PFR (Power Factorization Reprojection) [25], and MPI (Many Perspective Images) [26] are introduced for comparison. The results are shown in Table 1.

It can be seen from Table 1 that under the current calculation conditions, the camera orientation accuracy of this method is better than several other methods. This is related to the selection of the original image pair based on the characteristics of the monitoring camera’s trajectory and the removal of the unsuitable image for reconstruction before the bundle adjustment.

Another comparative experiment has been done to verify that the registration method proposed in this paper is better than these methods based on 3D feature point extraction in the process of lunar soil collection area’s point clouds registration. FPFH (Fast Point Feature Histograms) [27], Thrift [28], and TriSI [29] are three common 3D feature point extraction methods, all of them have good performance on point clouds registration. Obtain matching pairs between C0 and C1 by using each method mentioned above, and estimate the rotation matrix and translation vector with RANSAC and ICP. Use two indicators to measure registration accuracy [29]. The first indicator is the error between the estimated rotation matrix $R_{E}^{ij}$ and the reference rotation matrix $R_{GT}^{ij}$, which is defined as:

(6) $$\varepsilon_{r}^{ij}=\arccos\left(\frac{\mathrm{tr}\left(R_{GT}^{ij}{\left(R_{E}^{ij}\right)}^{-1}\right)-1}{2}\right)\cdot \frac{180}{\pi }$$

The second indicator is the error between the estimated translation vector $T_{E}^{ij}$ and the reference translation vector $T_{GT}^{ij}$, which is defined as:

(7) $$\varepsilon_{t}^{ij}=\frac{\left\|T_{GT}^{ij}-T_{E}^{ij}\right\|}{d_{res}}$$

*d_res_* = 1 mr, and is the average data resolution of the point cloud. The reference value is obtained by manual registration and ICP optimization. If the rotation error is less than 3° and the translation error is less than 3 mr, the registration result is considered successful. The simulation acquisition experiment has been done 35 times, the registration success rate is as follows:

It can be seen from Table 2 that this paper’s method has higher success rate in the process of lunar soil collection area’s point clouds registration. In fact, the reconstruction from 2D images to 3D model is a process of information loss. This paper’s registration method adds images into analysis objects, and exploits much more information. That’s the reason why it can perform better when many adverse effects exists caused by changes in the soil collection area, reconstruction errors, and other factors.

These two parts respectively ensure the accuracy of point clouds’ reconstruction and registration. Next, the collected soil’s volume can be calculated by point cloud comparison according to the design method in Section 3.2.

### 4.2. Collected Soil’s Volume Calculation

**Figure 11:**
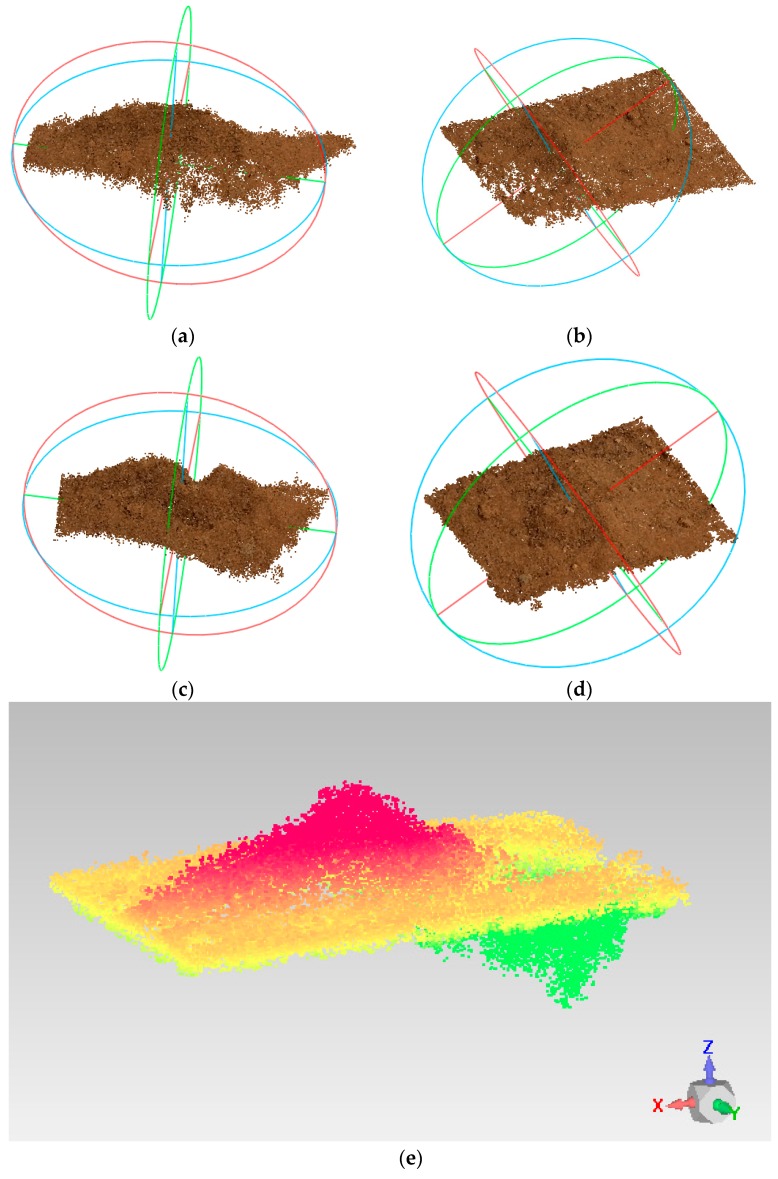
Reconstruction and comparison results before and after soil collection. (**a**) Before collection (horizontal view); (**b**) Before collection (vertical view); (**c**) After collection (horizontal view); (**d**) After collection (vertical view); (**e**) Results of point cloud comparison for area D in the gravity direction.

The reconstruction results are shown in Figure 11. In accordance with the comparison method described above, the changes in the reconstructed dense point clouds were calculated in the gravity direction before and after soil collection, as shown in Figure 11e, in which red represents sag, green represents bulge, and yellow represents no change at the current node.

**Figure 12:**
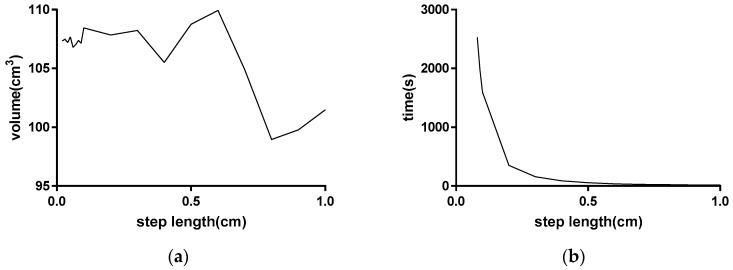
Trends of the obtained value and time cost based on different sampling steps. (**a**) Relationship between step length and accuracy; (**b**) Relationship between step length and computation speed.

It is difficult to choose the appropriate resampling step length parameters *s* when calculating the collection volume. A large step length is not beneficial to the precision of the calculation, and a small step will greatly increase the number of required calculations. A step length selection experiment was conducted to reflect the changes in the measurement accuracy and time cost based on different step lengths based on a true value of 107.4 cm^3^. Figure 12 shows that the measured values converge to the true value and the calculation time increases as the step size decreases. Table 3 shows that when the step size is less than 0.2 cm, the change in the measured value is small, but the time cost rapidly changes. Therefore, the value close to 0.2 cm can be selected as the resampling step under the above simulation conditions, and the obtained value is close to the true value based on this step length.

The method of trying different sampling steps one by one may be cumbersome in calculation. In fact, the optimal sampling step value can be estimated by calculating the ground sample distance (GSD) [30]. The GSD is the actual spatial distance of two adjacent pixels on the image, and it is a measure of limitation to image resolution. There is an online calculation method to estimate GSD. Use feature matching links to obtain several feature points on one image and their correspondent spatial points. Select two points from these feature points. The two points’ image distance is set to p (pixel), and the corresponding spatial distance is set to d (m), then p/d (m) can be treated as an approximation of GSD. The GSD is calculated for five times and the average value is taken as the step length. In this experiment, the calculation result is 0.186 cm. As can be seen from Figure 12, when this value is selected as step length, ideal measurement results can be obtained. The actual measured value is 107.79 cm^3^.

To verify the accuracy of the method, soil collection experiments were simulated with collection shovels of different sizes, and the results are shown in Table 4. The results indicate that the relative precision of the method is better than 1%, and this meets the needs of the collected soil’s volume measurement.

## 5. Conclusions

The monitoring camera fixed on the acquisition arm of the lunar lander can move flexibly and obtain measurement area images from multiple angles and positions. Using images taken from different angles in the soil collection area, the surface of this area can be reconstructed, and the required soil collection volume can be calculated. This paper proves that it is feasible to use a single monitoring camera to measure the amount of collected soil, and a measurement method is developed based on multiview reconstruction and dense point cloud comparison for a single camera. The paper proposes an accurate orientation method for sequences of images and calculates the position and orientation information of each camera view to fully and precisely reconstruct the dense point cloud of the surface of the area. Moreover, a strategy for registration and comparison between bi-temporal point clouds is proposed, and the change in area before and after soil collection, which equals the volume of collected soil, is calculated by summing the differences in point clouds in the direction of gravity. The experiments indicate that the results obtained using this method are consistent with the actual data, and the relative precision can reach 1%; therefore, the method meets the requirements of actual measurement tasks.

## Figures and Tables

**Table 1:** Comparison of different orientation methods’ re-projection error.

	SFM	PFR	MPI	This Paper’s Method
Re-projection error (pixel)	0.79	0.43	0.51	0.37

**Table 2:** The success rate of different registration methods.

	FPFH	Thrift	TriSI	This Paper’s Method
Success rate (%)	51.28	31.4286	62.85	88.57

**Table 3:** Partial measurement values and processing time for different sampling steps.

**Step/cm**	1.0	0.8	0.7	0.6	0.5	0.4	0.2	0.1	0.08
**Value/cm^3^**	101.48	98.95	104.89	109.93	108.76	105.5	107.84	108.44	107.58
**Time/s**	15.4	22.6	29.4	39.2	55.9	89.6	350.1	1595.1	2529.7

**Table 4:** Accuracy verification experiment with collection shovels of different sizes.

Actual Value/cm^3^	Measured Value/cm^3^	Error/cm^3^	Relative Precision
53.3	53.59	0.29	0.54%
88.6	88.76	0.16	0.18%
107.4	107.79	0.39	0.36%
116.3	115.87	0.43	0.37%
191.9	191.02	0.88	0.46%

## References

[1] Li C., Xie Z., Li Y., Liu H. (2013). A Novel End-Effector for Lunar Sample Acquisition and Return. Robot.

[2] Liang B., Ying H.E., Zou Y., Yang J., School S. (2016). Application of Time-of-Flight Camera for Relative Measurement of Non-Cooperative Target in Close Range. J. Astronaut..

[3] Zhang S., Xu Y., Liu S., Yan D. (2015). Calibration of Chang’e-3 Lunar Rover Stereo-camera System Based on Control Field. Geomatics Inf. Sci. Wuhan Univ..

[4] Zhang H., Zhang L. (2009). Binocular stereo matching algorithm for 3-D point cloud acquisition. J. Nanjing Univ. Aeronaut. Astronaut..

[5] Ding B., Wei Z., Ling S. (2015). An efficient method for organizing unordered images in 3D reconstruction. Optik.

[6] Fischler M.A., Bolles R.C. (1981). Random sample consensus: A paradigm for model fitting with applications to image analysis and automated cartography. Commun. ACM.

[7] Mikolajczyk K., Tuytelaars T., Schmid C., Zisserman A., Matas J., Schaffalitzky F., Kadir T., van Gool L. (2005). A comparison of affine region detectors. Int. J. Comput. Vis..

[8] Zhu Z., Guan B., Zhang X., Li D., Yu Q. (2014). Automatic Three-Dimensional Measurement of Large-Scale Structure Based on Vision Metrology. Sci. World J..

[9] Horn B.K.P. (1990). Relative orientation. Int. J. Comput. Vis..

[10] Stewénius H., Engels C., Nistér D. (2006). Recent developments on direct relative orientation. ISPRS J. Photogramm. Remote Sens..

[11] Pan H. (1999). A Direct Closed-Form Solution to General Relative Orientation of Two Stereo Views. Digit. Signal Process..

[12] Cronk S., Fraser C., Hanley H. (2006). Automated metric calibration of colour digital cameras. Photogramm. Rec..

[13] Lepetit V., Moreno-Noguer F., Fua P. (2009). EPnP: An accurate O(n) solution to the PnP problem. Int. J. Comput. Vis..

[14] Hesch J.A., Roumeliotis S.I. A Direct Least-Squares (DLS) method for PnP. Proceedings of the 2011 International Conference on Computer Vision ICCV.

[15] Hartley R., Zisserman A. (2003). Multiple View Geometry in Computer Vision.

[16] Bleyer M., Rhemann C., Rother C. (2011). Patch Match Stereo-Stereo Matching with Slanted Support Windows. Br. Mach. Vis. Conf..

[17] Zhu Z., Stamatopoulos C., Fraser C.S. (2015). Accurate and occlusion-robust multi-view stereo. ISPRS J. Photogramm. Remote Sens..

[18] Horaud R., Dornaika F. (2011). Hand-Eye Calibration. Int. J. Robot. Res..

[19] Pang S., Hu X., Cai Z., Gong J., Zhang M. (2018). Building Change Detection from Bi-Temporal Dense-Matching Point Clouds and Aerial Images. Sensors.

[20] Sharp G.C., Lee S.W., Wehe D.K. (2002). ICP Registration Using Invariant Features. IEEE Trans. Pattern Anal. Mach. Intell..

[21] Mackiewicz A., Ratajczak W. (1987). Principal component analysis. Chemom. Intell. Lab. Syst..

[22] Bender C.M., Mead L.R. (1995). D-dimensional moments of inertia. Am. J. Phys..

[23] Gargallo P., Prados E., Sturm P. Minimizing the Reprojection Error in Surface Reconstruction from Images. Proceedings of the International Conference on Computer Vision.

[24] Crandall D.J., Owens A., Snavely N., Huttenlocher D.P. (2013). SfM with MRFs: Discrete-Continuous Optimization for Large-Scale Structure from Motion. IEEE Trans. Pattern Anal. Mach. Intell..

[25] Xu L., Wang F., Fang H., Wen T. (2008). 3D reconstruction with minimum reprojection error using PFR. J. Huazhong Univ. Sci. Technol. (Nat. Sci.).

[26] Martinec D., Pajdla T. (2002). Structure from Many Perspective Images with Occlusions. Computer Vision—ECCV 2002.

[27] Rusu R.B., Blodow N., Beetz M. Fast point feature histograms (FPFH) for 3D registration. Proceedings of the IEEE International Conference on Robotics and Automation.

[28] Flint A., Dick A., Van Den Hengel A. Thrift: Local 3D Structure Recognition. Proceedings of the Biennial Conference of the Australian Pattern Recognition Society on Digital Image Computing Techniques and Applications.

[29] Guo Y., Sohel F.A., Bennamoun M., Lu M., Wan J. TriSI: A Distinctive Local Surface Descriptor for 3D Modeling and Object Recognition. Proceedings of the International Conference on Computer Graphics Theory and Applications.

[30] Felipe-García B., Hernández-López D., Lerma J.L. (2012). Analysis of the ground sample distance on large photogrammetric surveys. Appl. Geomat..

